# Large Primary Transethmoidal Encephalocele Presenting in an Adult

**DOI:** 10.7759/cureus.16122

**Published:** 2021-07-02

**Authors:** Jordan Lam, Darrin J Lee, Azeem Oladunjoye

**Affiliations:** 1 Neurological Surgery, University of Michigan, Ann Arbor, USA; 2 Neurological Surgery, University of Southern California, Los Angeles, USA; 3 Department of Surgery, San Joaquin General Hospital, French Camp, USA

**Keywords:** encephalocele, adult, nontraumatic, congenital, cribriform plate, cerebrospinal fluid rhinorrhea, surgery

## Abstract

Transethmoidal encephaloceles are rare and most commonly present at birth with congenital abnormalities, cerebrospinal fluid rhinorrhea, or visual symptoms. Here, we report the case of a 43-year-old presenting with longstanding headache, blurry vision, anosmia, and rhinorrhea. Magnetic resonance imaging confirmed a transethmoidal encephalocele. The patient underwent craniotomy for resection of the encephalocele and repair of the cribriform defect. The postoperative course was uneventful, and the patient was discharged home with the resolution of rhinorrhea and headache. This report highlights a rare case of primary transethmoidal encephalocele undiagnosed until adulthood despite longstanding symptoms and successful treatment with resolution of symptoms.

## Introduction

Encephaloceles are herniations of brain tissue through a skull defect. Comprising only 1-3% of encephaloceles, transethmoidal encephaloceles are among the rarest [1,2]. While transethmoidal encephaloceles can be secondary to trauma or a cranial lesion, the majority are congenital, caused by a failure of neural tube closure, and present at birth with craniofacial abnormalities. Those undiagnosed at birth can present with nasal obstruction, visual symptoms, or cerebrospinal fluid (CSF) rhinorrhea [1,3]. If untreated, the latter can lead to complications, including recurrent meningitis, before a diagnosis is made [1,3,4].

In this case report, we describe a rare case of primary transethmoidal encephalocele, undiagnosed until adulthood despite longstanding symptoms and successful treatment with resolution of symptoms.

## Case presentation

**Figure 1:**
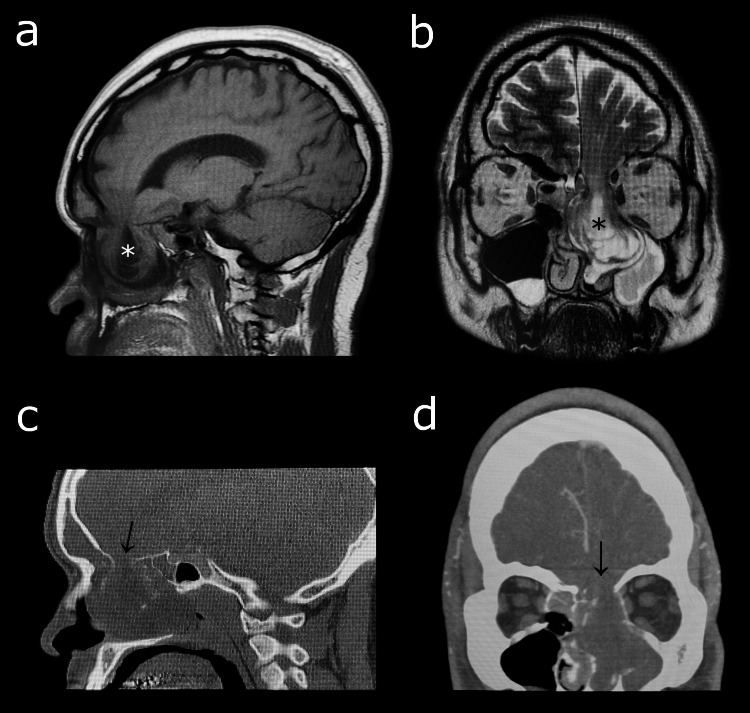
Imaging findings. A 43-year-old male presented with a lifelong history of headache, blurry vision, anosmia, and rhinorrhea. CT and MRI showed a 36 × 35 × 33 mm encephalocele herniating into the left ethmoids and nasal fossa with bony remodeling. (a) Sagittal T1 MRI and (b) coronal T2 MRI showing the encephalocele (*). (c) Sagittal CT and (d) coronal CT showing the bony defect (arrows). The encephalocele was resected and the cribriform plate was reconstructed. The patient was discharged with no recurrence of symptoms. CT: computed tomography; MRI: magnetic resonance imaging

A 43-year-old male with a body mass index (BMI) of 35 presented with a lifelong history of headache, blurry vision, anosmia, and rhinorrhea, but no reported history of significant trauma. The patient was previously worked up for bacterial sinusitis and allergic rhinitis and underwent unsuccessful trials of antibiotics, antihistamines, and intranasal steroids. The examination was remarkable for anosmia and visual impairment but no craniofacial abnormalities. Computed tomography and magnetic resonance imaging showed a 36 × 35 × 33 mm encephalocele herniating into the left ethmoids and nasal fossa with bony remodeling. There was complete opacification of the left frontal sinus and near-complete opacification of bilateral maxillary sinuses (Figure 1).

The patient underwent bifrontal craniotomy. The encephalocele was found to herniate through a defect in the left cribriform plate and communicate with the left frontal sinus. The encephalocele was resected and the cribriform plate was reconstructed with split-thickness autograft and titanium mesh. Exenteration and cranialization of the frontal sinus were performed bilaterally and the frontal sinus ostia were plugged with an abdominal fat graft. Both an external ventricular drain and lumbar drain were inserted to divert CSF while the repair healed. The postoperative course was uneventful with no signs of CSF leak and the patient was successfully discharged on postoperative day nine with no recurrence of symptoms. The patient remained well and symptom-free at the six-month follow-up.

## Discussion

This is a rare case of a patient with a large primary (congenital) transethmoidal encephalocele that presented for the first time at 43 years of age. Despite the lack of craniofacial abnormalities, the absence of reported significant trauma history, lifelong duration of symptoms, and bony remodeling changes on imaging are highly suggestive of a congenital etiology. Additionally, no lesions suspicious for malignancy were present on imaging, which may also cause a secondary encephalocele [5].

Those undiagnosed at birth can present with nasal obstruction, visual symptoms, or rhinorrhea [1,3]. If untreated, the latter can lead to recurrent meningitis before a diagnosis is made [1,3,4]. Notably, in this case, the patient did not experience meningitis as a sequela of CSF rhinorrhea in contrast to the majority of previous reports of transethmoidal encephalocele presenting in adulthood [4,6]. However, the patient did experience a lifetime of symptoms, incorrect diagnoses, and unsuccessful treatments, representing an enormous burden on quality of life and medical costs. Therefore, prompt repair is emphasized to improve symptoms and prevent secondary meningitis.

An interesting link exists between obesity, idiopathic intracranial hypertension, and spontaneous CSF leak of the anterior skull base which may result in an encephalocele [7-9]. In this case, the patient was obese, with a BMI of 35, and presented with similar symptoms to a patient with idiopathic intracranial hypertension and CSF leak. Therefore, despite the chronic nature of symptoms, a longstanding spontaneous CSF leak secondary to idiopathic intracranial hypertension could not be ruled out as a possible etiology in this patient. Additionally, obesity has been associated with higher rates of postoperative CSF leak [7,8,10]. Considering this in addition to the size of the defect and the encephalocele, external ventricular and lumbar drains were used postoperatively in this patient to divert CSF while the repair healed.

Endoscopic endonasal approaches have become the mainstay of treatment for CSF leaks and encephaloceles in the anterior skull base, with closure rates exceeding 90% [10-12]. However, given the size of this patient’s encephalocele, an endonasal approach alone was not sufficient and the patient was treated with an open bifrontal approach. An additional indication for an open approach in this case was the consideration of the chronic mucocele in the left frontal sinus, requiring exenteration and cranialization. Following resection of the encephalocele, the cribriform plate was reconstructed with split-thickness autograft and titanium mesh. Titanium mesh was used in this patient to best secure the autologous free bone graft to the anterior skull base and provide a more esthetic reconstruction.

## Conclusions

In conclusion, longstanding rhinorrhea accompanied by symptoms such as anosmia, headache, and visual abnormalities should prompt investigation into anterior skull base abnormalities. In the case of transethmoidal encephalocele, prompt surgical repair is essential to improve symptoms and prevent complications such as recurrent meningitis.
